# Visceral Hypersensitivity Is Provoked by 2,4,6-Trinitrobenzene Sulfonic Acid-Induced Ileitis in Rats

**DOI:** 10.3389/fphar.2016.00214

**Published:** 2016-07-22

**Authors:** Manoj K. Shah, Juan Wan, Habibullah Janyaro, Adnan H. Tahir, Luying Cui, Ming-Xing Ding

**Affiliations:** College of Veterinary Medicine, Huazhong Agricultural UniversityWuhan, China

**Keywords:** TNBS, cytokines, ileitis, visceral hypersensitivity, visceromotor response, crohn’s disease, calcitonin gene-related peptide, rat

## Abstract

**Background and Aims:** Crohn’s Disease (CD), a chronic Inflammatory Bowel Disease, can occur in any part of the gastrointestinal tract, but most frequently in the ileum. Visceral hypersensitivity contributes for development of chronic abdominal pain in this disease. Currently, the understanding of the mechanism underlying hypersensitivity of Crohn’s ileitis has been hindered by a lack of specific animal model. The present study is undertaken to investigate the visceral hypersensitivity provoked by 2,4,6-trinitrobenzene sulfonic (TNBS)-induced ileitis rats.

**Methods:** Male Sprague-Dawley rats were anaesthetized and laparotomized for intraileal injection of TNBS (0.6 ml, 80 mg/kg body weight in 30% ethanol, *n* = 48), an equal volume of 30% Ethanol (*n* = 24), and Saline (*n* = 24), respectively. Visceral hypersensitivity was assessed by visceromotor responses (VMR) to 20, 40, 60, 80, and 100 mmHg colorectal distension pressure (CRD) at day 1, 3, 7, 14, 21, and 28. Immediately after CRD test, the rats were euthanized for collecting the terminal ileal segment for histopathological examinations and ELISA of myleoperoxidase and cytokines (TNF-α, IL-1β, IL-6), and dorsal root ganglia (T_11_) for determination of calcitonin gene-related peptide by immunohistochemistry, respectively.

**Results:** Among all groups, TNBS-treatment showed transmural inflammation initially at 3 days, reached maximum at 7 days and persisted up to 21 days. The rats with ileitis exhibited (*P* < 0.05) VMR to CRD at day 7 to day 21. The calcitonin gene-related peptide-immunoreactive positive cells increased (*P* < 0.05) in dorsal root ganglia at day 7 to 21, which was persistently consistent with visceral hypersensitivity in TNBS-treated rats.

**Conclusion:** TNBS injection into the ileum induced transmural ileitis including granuloma and visceral hypersensitivity. As this model mimics clinical manifestations of CD, it may provide a road map to probe the pathogenesis of gut inflammation and visceral hypersensitivity, as well as for establishing the therapeutic protocol for Crohn’s ileitis.

## Introduction

Crohn’s disease, a major form of human IBDs, is a chronic, remitting, and relapsing inflammatory disorder of gastrointestinal tract that can affect animal and almost all ages of human population. It leads to a wide range of symptoms, such as diarrhea, nausea, abdominal pain, and VH, which has a significant effect on the life quality of affected patients (Wagtmans et al., 1998; Simren et al., 2002; Faure and Giguere, 2008). In IBD, the continuous release of inflammatory mediators can result enteric nerve endings sensitization and abdominal pain in 70% of IBD patients, which is not only present during acute flares of disease but also during remission (Minderhoud et al., 2004; Bielefeldt et al., 2009). Two thirds of CD manifest as a transmural inflammation in the terminal ileum. Whether CD patients suffer from visceral sensitivity disturbances seem somewhat controversial, and its evidence is likewise scarce. Bernstein et al. (1996) discovered that patients with ileal CD had reduced sensitivity and autonomic reflex responses when the rectum was distended. However, Faure and Giguere (2008) reported that CD patients coexisting functional GI disorders on rectal distension showed an increased rectal hypersensitivity. The discrepancy regarding VH should be addressed properly using CD model as a choice to add the knowledge in this filed. Over the years, most studies have investigated VH utilizing colitis models (Diop et al., 2002; Zhou et al., 2008; Feng et al., 2012). However, the colitis could not be appropriate for studying the precise mechanism of VH accompanying CD because its location, duration, microbiota and central regulating mechanism are different from the CD of ileal origin. So far no ileitis model that provokes VH has been reported.

Experimental models for intestinal inflammations were commonly induced by TNBS, oxazolone, and dextran sodium sulfate. Among them, TNBS is diluted in ethanol to disrupt the mucosal barrier. It invades and causes haptenization of colonic mucosal proteins (Neurath et al., 2000), and has primarily been regarded as a model for studying CD and UC (Wallace et al., 1995; Jurjus et al., 2004). Over the years, few studies used TNBS to induce ileitis for studying the pathophysiology of ileal diseases. Nurgali et al. (2011) injected TNBS solution into the ileal lumen of guinea pigs for studying the morphological and functional changes in neurons projecting to the ileal mucosa at the early stage after inflammatory damage. Sjogren et al. (1994) and Goldhill et al. (1999) used TNBS-induced ileitis for investigation of *in vivo* ileal myoelectric responses to acute enteric inflammations in rabbits and *in vitro* smooth muscle contractility in rats. So far as we know, there is no report on VH in ileitis models.

Crohn’s disease is characterized by the infiltration of inflammatory and immune cells (e.g., mast cells, neutrophils, *T*-lymphocytes, and macrophages) that interact and release certain enzymes, e.g., MPO and cytokines etc. Intestinal specimens biopsied from Crohn’s patient have shown a transmural chronic inflammation with upregulated MPO and cytokines (e.g., TNF-α, IL-1β, and IL-6) (Ligumsky et al., 1990; Gross et al., 1992; Reinecker et al., 1993; O’Hara et al., 2007). These cytokines are believed to result in neurogenic inflammation (the release of substance-P, CGRP, and nerve growth factor), which as a consequence influences neurotransmission, muscle contractility and secretory functions, and abdominal pain (Collins et al., 1996; Stein et al., 1998). Cytokines can be used to grade the severity of TNBS-colitis in rats (Strober and Fuss, 2011; Du et al., 2014). The study by Adam et al. (2006) looked for the association between the severity of colitis with the levels of serum IL-6 and VMR, and suggested inflammatory changes and cytokine levels are responsible for initiation and maintenance of VH. Therefore, the assessment of cytokines and CGRP level in ileitis leading to hypersensitivity seem imperative to understand the pathogenesis of CD as well as for evaluating the effectiveness of potent therapeutic agents.

In the present study, to investigate the VH provoked by ileitis, TNBS/ethanol solution was injected into the terminal ileum in rats. The DAI, macroscopic and microscopic damage scores, CGRP level, MPO level, cytokines levels, and VMR to colorectal distention were assessed to explore the mechanism underlying VH in ileitis.

## Materials and Methods

### Experimental Animal

Ninety six male Sprague–Dawley rats, aged 5–6 weeks and weighing 190–289 g, were used for the experiments. The rats were purchased from Hubei Provincial Center for Experimental Animal Research, Wuhan, and housed in temperature controlled room with a 12 h dark–light cycle and provided free access to standard feed and tap water. The rats were acclimatized to laboratory environment for 1 week before initiation of the experiment. All experiments involving live rats were performed according to the stipulated rules for experimental usage of laboratory animals (the regulation of the administration of affairs concerning experimental animals of P.R. China). All protocols were approved by the Laboratory Animal Research Center of Hubei province and the ethics committee of Huazhong Agricultural University (Permit number: HZAUMO-2015-12).

### Induction of Ileitis

Rats were randomly assigned for 48 in TNBS group, 24 in ethanol group and 24 in saline group. After fasted for 24 h, the rats were anesthetized with Pentobarbital (50 mg/kg body weight, Gbcbio Technologies Inc., China) intraperitoneally. Following the aseptic surgical procedure, the midventral laparatomy was performed. The terminal part of the ileum was exteriorized gently on moist sterile gauze. The various combinations of TNBS (60–100 mg/kg body weight, Sigma Aldrich, USA) and ethanol (20–40%) were tested in our pretest. The dose of TNBS (0.6 ml, 80 mg/kg body weight in 30% ethanol) was considered suitable to induce the moderate ileitis according to DAI, macroscopic and microscopic damage scores, and MPO level. In the formal experiment, 0.6 ml TNBS (80 mg/kg body weight in 30% Ethanol), an equal volume of 30% Ethanol and 0.9% NaCl were injected into the rat’s ileal lumen 10 cm proximal to the ileo-cecal junction for TNBS-, ethanol- and saline-groups, respectively, using a 30-gauge needle fitted to 1 ml syringe. Before injection, a portion of ileum extending 3.5 cm orad and 3.5 cm aborad to the injection site was isolated, and milked free of luminal contents. To keep the injected chemicals to fully contact with the ileal mucosa, the cranial and caudal sides of the injected ileal fragment were occluded for 5 min with thumb and forefingers with mild pressure (which just prevented the flow of the injected solution, but not caused intestinal ischemia judged by its color), and were marked by tying two fine silk ligatures (5–0) loosely in the mesentery for later location. Then the ileum was returned to the cavity. The peritoneum along with linea alba was sutured with catgut 4–0 in simple interrupted pattern. Before the suture of skin, 0.5% Bupivacaine HCl (Shandong Hualu Pharamaceutical Co. Ltd., China) was instilled on the incised wound. Finally, the skin was sutured using silk 5–0 in simple interrupted pattern. Upon completion of the surgery, the rats were placed in a rat box. After recovery from anesthesia, the rats were allowed to access food and water *ad libitum*. The operated rats were checked daily and their wound received pyrrolidone solution (Jiaozuo Zhongwei Chemical Co., Ltd., China) application twice a day till healing.

### Visceromotor Response to Colorectal Distension

**FIGURE 1:**
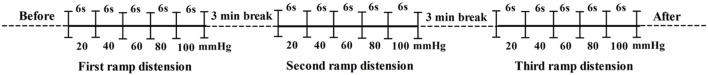
**Scheme for CRD and EMG**.

The VMR to CRD was used as an objective indicator of VH. The present study used EMG to quantify the magnitude of the abdominal contractions evoked by CRD. The EMG was recorded between 8 a.m. and 1 p.m. at day 1, 3, 7, 14, 21, and 28 after the injection as described previously (Winston et al., 2007). Briefly, the rats were brought to the testing room, and acclimatized to the testing environment for 3 days. A balloon (5-cm long) which was constructed with the finger of a latex glove attached to a flexible tube was inflated and left overnight to make the latex stretched and the balloon compliant. The caudal midventral region of the rats were clipped, shaved, and painted with pyrrolidone solution. The rats were placed in a plastic cylinder with a sound-attenuating and dark chamber to limit their movement and allowed to be acclimatized for 30 min before testing. The flexible balloon was lubricated with paraffin and inserted 8 cm into the descending colon and rectum via the anus and held in place by taping the tube to the tail. The tube of the balloon was connected via a Y-connector to a 20 ml syringe for inflating balloon and a sphygmomanometer for measuring the distension pressure. Two thirty-two-gauge nickel-steel needles (0.25 mm in diameter and 25 mm in length, Suzhou Medical Supplies, Co. Ltd., China) as EMG electrodes were inserted via the skin into abdominal muscle, placed 2–3 cm apart just before CRD and EMG recording. Then both electrodes along with skin were clamped and connected to the EMG-machine (Nanjing Medease Science and Technology Co. Ltd., China). The balloon was inflated every 6 s in a continuous ramp distension mode (20, 40, 60, 80, and 100 mmHg) and then a 3 min break was maintained before another set of distension (**Figure 1**). The procedure of balloon distension and EMG recordings were repeated three times. A software (MedLab-U/4C501) was used to quantify the magnitude of EMG signals and mechanical irritation signals. VMRs to CRD were quantified as the total area of electromyographic activity during balloon inflation minus the resting activity and were presented as millivolt per-second (mV/s).

### Symptoms and Disease Activity Index

Symptoms such as body weight, water/food consumption, mortality, stool consistency, and bloody feces were observed at day 0, 1, 3, 7, 14, 21, and 28. The mean body weights at day 0 and at sampling days were expressed as BW_0_ and BW_n_, respectively. The percentage change in the body weight was calculated as follows: Δ (%) = (BW_n_ – BW_0_)/BW_0_ × 100%. Quantification of DAI comprised body weight (0–4), stool consistency (0–4) and rectal bleeding (0–4), and was determined daily between 8 and 10 a.m. as described in detail elsewhere (Cooper et al., 1993; Siegmund et al., 2001). The scores from these three parameters were summed up as DAI ranging from 0 (healthy) to 12 (maximal severity) (Alex et al., 2009).

### Tissue Sampling

Sixteen rats taken from the TNBS group (8 rats), the ethanol group (4 rats) and the saline group (4 rats) at day 1, 3, 7, 14, 21, and 28, respectively, were euthanized immediately after CRD test. The 10-cm terminal ileum of each rat was excised. After being washed in phosphated buffer saline solution, two ileal segments (one 5 mm, another 1 cm long) in sequence were taken from the distal end of each ileum. The short segment was fixed in 4% buffered formaldehyde for histopathological examination. The longer segment was weighted, frozen in liquid nitrogen and transferred to store at -80°C for assaying the concentrations of MPO, TNF-α, IL-1β, and IL-6. The DRG of T_11_ thoracic (left and right) enlargement was dissected out properly and post-fixed in 4% buffered formaldehyde for immunohistochemical analysis of CGRP.

### Macroscopic and Microscopic Observation

The macroscopic appearance was scored by two blinded investigators on a scale of 0–4, described by Morris et al. (1989). Ileal tissues fixed in 4% buffered formaldehyde were embedded in paraffin. The tissue block was sectioned at a thickness of 5-μm, and mounted on poly lysine coated slides. Six serial slides were deparaffinized and rehydrated sequentially. Three slides were stained with haematoxylin and eosin (H&E) and another three stained with 0.5% toluidine blue. The slides were visualized with the images under a light microscope (Nikon Eclipse 80I, Nikon Corporation, and Tokyo, Japan) with a 20 × objective lens. Microscopic changes were blindly assessed by two investigators using the scoring system on 0–9 scales as previously described (Du et al., 2014). Mast cells were counted in three fields per ileal section, and the average number of mast cells per field was calculated.

### CGRP Immunohistochemistry in DRG

The DRG fixed in 4% buffered formaldehyde was embedded in paraffin. The tissue block was sectioned at a thickness of 4-μm, and mounted on poly lysine coated slides. Four serial slides were deparaffinized and rehydrated sequentially, followed by the Streptavidin–Biotin Complex immunohistochemistry procedure as follows. The serial slides were incubated with rabbit-anti-CGRP (Abcam Inc., Cambridge, MA-02139-1517, USA; 1:100 diluted in PBS), and PBS (the negative control), respectively, and, sequentially, for 24 h at 4°C. Then, they were treated with secondary antibodies (SA1022-anti-rabbit IgG Kit, Wuhan Boster Biological Technology Ltd., Wuhan, China). Avidin-biotin complex Staining (Wuhan Boster Biological Technology Ltd., Wuhan, China) was visualized with diaminobenzidine (DAB) for 1 min at room temperature. All sections were dehydrated in graded ethanol series, made transparent in xylene, coverslipped, and air-dried. The slides were visualized with the images of the stained areas under a light microscope (Nikon ECLIPSE 80I, Nikon Corporation, and Tokyo, Japan), and 3 fields of each slide were observed with a 20 × objective lens. The numbers of CGRP positive cells were counted with the Image-Pro plus 6.0 system (Media Cybernetics, Inc., Bethesda, MD, USA). The mean values calculated from three sections represented the CGRP positive cells per rat.

### Measurement of MPO and Cytokines

The ileal tissue was grinded and homogenized in 1 ml PBS, pH 7.2 at 4°C. The solution was centrifuged at 5000 g at 4°C for 10 min. The protein concentration of the supernatant was determined using Nanodrop Spectrophotometer (Thermo Fisher Scientific, Inc., USA). The MPO concentration was assayed with MPO ELISA Kit (eBioscience, Inc., San Diego, CA 92121, USA) according to the manufacturer’s instructions. Likewise, the concentrations of TNF-α, IL-1β, and IL-6 were measured using ELISA kits (NeoBioscience, China). Each sample was analyzed in triplicate and the values were presented as pg/mg.

### Statistical Analysis

All data were expressed as the mean ± SD. Statistical analysis was performed using SPSS version 18 (SPSS Inc., Chicago, IL, USA). The statistical comparisons for parametric data (Body Weight, CGRP, MPO, TNF-α, IL-1β, and IL-6) were carried out using one-way analysis of variance (ANOVA) followed by Bonferroni *post hoc* test (Dunnett’s T3 test was used if variables were unequal). The statistical differences of non-parametric values (DAI, macroscopic and microscopic scores, mast cells and VMR) among groups were identified using Kruskal–Wallis ANOVA followed by the rank-based Mann–Whitney *U*-test. The VMR at 20, 40, 60, 80, and 100 mmHg each for 6 s was added to calculate the total VMR for 30 s. Correlation between total VMR and CGRP level was assessed by computing Pearson’s *P* as correlation coefficient. A difference was accepted as significant if *P* was less than 0.05.

## Results

### Symptom Observation and Disease Activity Index

**FIGURE 2:**
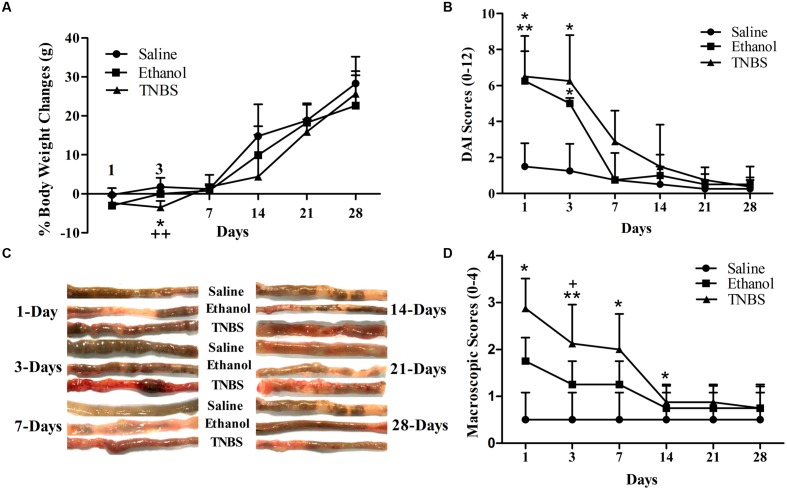
**Effects of TNBS administration on body weights and macroscopic pathologic changes in rats.** Body weight changes. ^∗^*P* < 0.05, TNBS group vs. Saline group; **^++^***P* < 0.01, TNBS group vs. Ethanol group; One-way ANOVA followed by Bonferroni’s post-test **(A)**. Scores of DAI ranging from 0 (healthy) to 12 (maximal severity of ileitis): body weight (0–4), stool consistency (0–4), and rectal bleeding (0–4) **(B)**. Macroscopic pathologic changes at day 1,3,7,14,21, and 28 **(C)**. Macroscopic change scores on a scale of 0–4 **(D)**. ^∗∗^*P* < 0.01, ^∗^*P* < 0.05; TNBS group/Ethanol group vs. Saline group. ^+^*P* < 0.05; TNBS group vs. Ethanol group. Kruskal–Wallis analysis followed by Mann–Whitney *U*-test.

There were no difference (*P* > 0.05) in body weight between the ethanol- or TNBS- rats and saline-rats at day 1. Compared with the saline-rats, the body weight in the ethanol- or TNBS- treated rats decreased (*P* = 0.031 or 0.001) at day 3 after the injection. But the body weight of the TNBS group was not different (*P* > 0.05) from that of the ethanol group at day 3. There was no difference (*P* > 0.05) in the body weight among three groups during day 7 to 28 (**Figure 2A**). The TNBS- or Ethanol-administrated rats displayed diarrhea, reduced grooming and sluggishness at day 1, bloody diarrhea at day 2 to 5 after the treatment. The rats showed normal fecal pellets at day 7. Compared with the saline group, the rats of ethanol groups showed higher (*P* = 0.018 and 0.013) DAI scores at day 1 and 3. The TNBS-treated rats showed increased (*P* = 0.005 and 0.013) DAI scores as compared to the saline group at day 1 and 3. There was no difference in the DAI scores between TNBS group and ethanol group during the experiment (**Figure 2B**).

### Macroscopic Scoring of Ileitis

The macroscopic examination of ileum showed no apparent change (*P* > 0.05) in the saline group during the experiment. The ethanol-treated ileum showed erythema, mucosal necrosis and edema in the injected segment of the terminal ileum at day 1, but no significant morphological changes were observed at day 3 and thereafter. The TNBS-treated ileum revealed pathologic changes as similar to the ethanol-treated ileum at day 1, and mild erythema, mucosal necrosis, adhesion with cecum or mesentery, ulcerations and the enteric wall thickness at day 3 and day 7, and no obvious pathological changes at day 14 to 28 (**Figure 2C**). No apparent change was observed in the adjacent intestinal segment such as jejunum, cecum, and colon. As shown in **Figure 2D**, no difference was found in the macroscopic scores between the saline group and ethanol group. TNBS-treated ileums revealed increased (*P* = 0.012) macroscopic scores as compared to ethanol group at day 1. The macroscopic scores in TNBS group were higher (*P* < 0.05) than those in the saline group at day 1 to 7. There was no difference (*P* > 0.05) in the macroscopic scores between the TNBS group and saline group at day 14 to 28.

### Microscopic Scoring of Ileitis

**FIGURE 3:**
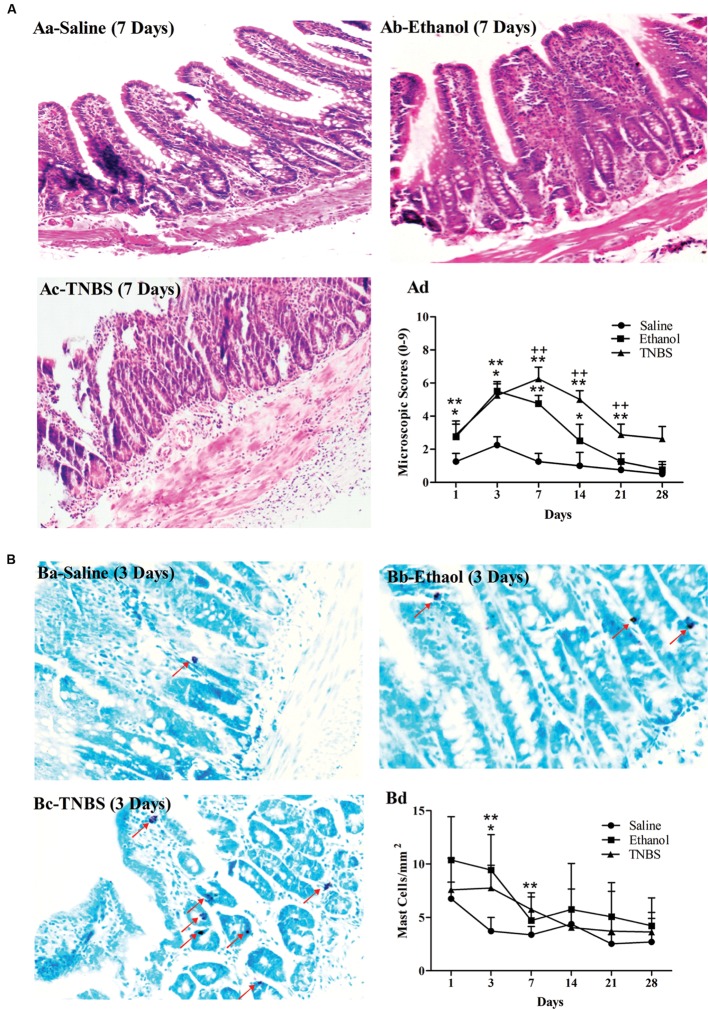
**Effects of TNBS administration on histopathology of the ileum in rats.** Microscopic changes of the ileum stained with hematoxylin and eosin (H&E) **(Aa–c)**. The microscopic change scores on a 0–9 scale, crypt architecture (0–3), inflammatory cell infiltration (0–3), and ulceration (0–3) **(Ad)**. The morphological changes of the ileum stained with toluidine blue **(Ba–c)**. The number of the toluidine-blue-stained positive mucosal mast cells in the ileum **(Bd)**. ^∗∗^*P* < 0.01, ^∗^*P* < 0.05; TNBS group/Ethanol group vs. Saline group. ^++^*P* < 0.01; TNBS group vs. Ethanol group. Kruskal–Wallis analysis followed by Mann–Whitney *U*-test.

The severity of inflammation was assessed according to crypt architecture, inflammatory cell infiltration and ulceration (**Figures 3Aa,b,c**). No apparent pathological changes were found in saline-injected ileums during the experiment. The ethanol-treated ileums revealed a moderate number of neutrophils in the lamina propria, minimal or moderate interstitial wall edema, reduced crypt depth, and decreased goblet cells while TNBS-treated ileums showed shorter crypt, disordered villi, debris of epithelium, and less goblet cells, neutrophils, lymphocytes, granulomas and severe edema in the submucosa at day 1 to 14. The pathological changes in the TNBS group abated at day 21, and were not obviously observed at day 28. Compared with the saline group, the ethanol-treated ileum showed higher (*P* < 0.05) microscopic scores at day 1 to 14 while the TNBS-treated ileum demonstrated increased microscopic scores (*P* < 0.01) at day 1 to 21. Compared with the ethanol group, the TNBS treatment resulted in higher (*P* < 0.01) microscopic scores at day 7 to 21. There was no difference (*P* > 0.05) in the microscopic score among three groups at day 28 (**Figure 3Ad**).

**Figures 3Ba,b,c** represent the mast cells observed in the ileum stained with toluidine blue at day 3. There were no differences (*P* > 0.05) in mucosal mast cells among three groups at day 1 and day 14 to 28. Compared with the saline group, the ethanol group showed more (*P* = 0.019) mucosal mast cells at day 3 while the TNBS treatment resulted in increased (*P* = 0.01 and 0.008) mucosal mast cells at day 3 and 7 (**Figure 3Bd**).

### The Expression Levels of CGRP in the DRG

**FIGURE 4:**
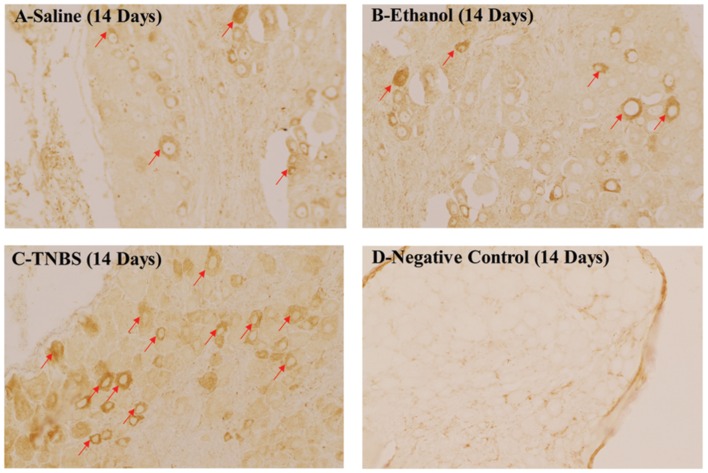
**Effects of TNBS administration on the expression levels of CGRP in the DRG of the rats.** Densities of GRP-IR cells in the DRG at day 14 **(A–C)**. The negative control at day 14 **(D)**.

The CGRP-IR cells in the DRG at day 14 have been shown in **Figures 4A–D**. The CGRP expressions in the DRG of TNBS-treated rats were higher than rats of saline or ethanol group. There was no difference in CGRP-IR positive cells (*P* > 0.05) among three groups at day 1 and 3. The CGRP-IR cells in TNBS-treated rats increased by 2.06-fold (*P* < 0.05) and 1.68-fold as compared to saline group and ethanol group, respectively, at day 7. In TNBS-treated rats, the CGRP-IR positive cells increased further and reached (*P* < 0.01) greatest, i.e., 5.56-fold and 4.26-fold as compared to saline group and ethanol group, respectively, at day 14. Thereafter, CGRP-IR positive cells in TNBS treated rats decreased but remained 1.88-fold and 1.57-fold higher (*P* < 0.01) as compared to saline group and ethanol group, respectively, at day 21. There was no difference in CGRP-IR positive cells (*P* > 0.05) among three groups at day 28 (**Table 1**).

**Table 1:** Effects of TNBS administration on CGRP-immunoreactivities expression in DRG of rats.

Group	Days					
	
	1	3	7	14	21	28
Saline	10.78 ± 3.29	14.27 ± 6.97	13.64 ± 5.08	11.18 ± 2.27	11.09 ± 1.82	9.74 ± 1.12
Ethanol	12.67 ± 4.95	16.59 ± 0.46	16.73 ± 8.92	14.61 ± 3.61	13.26 ± 2.36	9.95 ± 0.76
TNBS	16.54 ± 2.61	19.87 ± 5.18	25.52 ± 2.01^a^	44.02 ± 7.73^AB^	21.48 ± 2.22^AB^	11.28 ± 0.66


*Data are expressed as Mean ± SD. ^A^*P* < 0.01 and ^a^*P* < 0.05 represents significant difference between TNBS group and Saline group. **^B^***P* < 0.01 represents significant difference between TNBS group and Ethanol group. One-way ANOVA followed by Dunnett’s T3 post-test.*

### Concentrations of MPO and Cytokines

**FIGURE 5:**
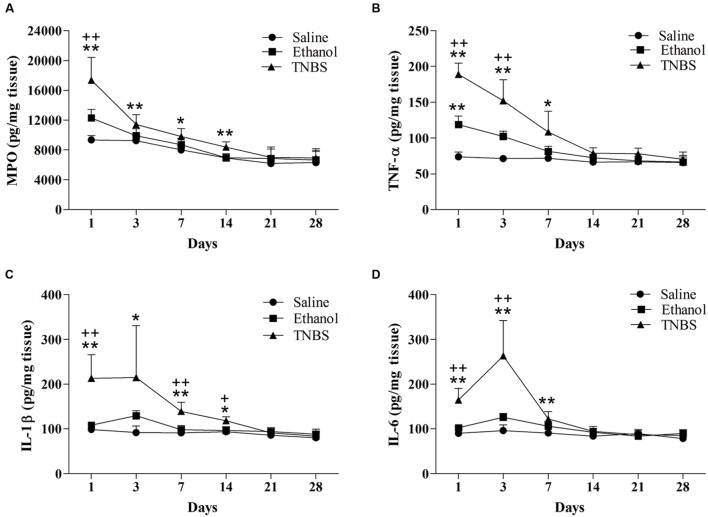
**Effects of TNBS administration on the levels of MPO **(A)**, TNF-α **(B)**, IL-1β **(C)**, and IL-6 **(D)** in the ileal tissue of the rats.** ^∗∗^*P* < 0.01, ^∗^*P* < 0.05; TNBS group/Ethanol group vs. Saline group. ^++^*P* < 0.01, ^+^*P* < 0.05; TNBS group vs. Ethanol group. (TNBS vs. Ethanol/Saline group; One-way ANOVA followed by Dunnett’s T3 post-test) (Ethanol group vs. Saline group; One-way ANOVA followed by Bonferroni’s post-test).

There was no difference in MPO concentration between the TNBS group and the ethanol group during the experiment. The MPO concentration in the ileal tissue of the TNBS group increased (*P* < 0.01) at day 1 to 14 compared with the saline group. Likewise, the TNBS-treated ileums showed (*P* = 0.005 and 0.01) elevated MPO concentration as compared to the ethanol-treated rats at day 1 and 14. There was no difference (*P* > 0.05) in MPO concentration among three groups at day 21 and 28 (**Figure 5A**).

As illustrated in **Figures 5B–D**, the ethanol group revealed increased (*P* = 0.001) TNF-α concentration as compared to the saline group at day 1. TNF-α concentration in the TNBS group was higher (*P* < 0.05) at day 1, 3, and 7 compared with the saline group, and higher (*P* = 0.000 and 0.005) at day 1 and 3 compared with the ethanol group. No significant differences in TNF-α concentration were observed (*P* > 0.05) among three groups at day 14 to 28. Compared with the saline group, the TNBS-treated rats showed increased (*P* < 0.05) IL-1β concentration at day 1 to 14. The TNBS group revealed elevated (*P* < 0.05) IL-1β concentration compared with the ethanol group at day 1, 7 and 14. The IL-1β concentration decreased (*P* > 0.05) among three groups at day 21 and 28. The TNBS-treated ileum revealed increased (*P* = 0.00, 0.001 and 0.002) IL-6 concentration compared with the saline group at day 1, 3, and 7. Rats in the TNBS group showed increased (*P* = 0.00 and 0.005) IL-6 concentration at day 1 and 3 compared with the ethanol rats. There was no difference in IL-6 concentration (*P* > 0.05) among three groups at day 14 to day 28.

### Visceromotor Response to Colorectal Distension

**FIGURE 6:**
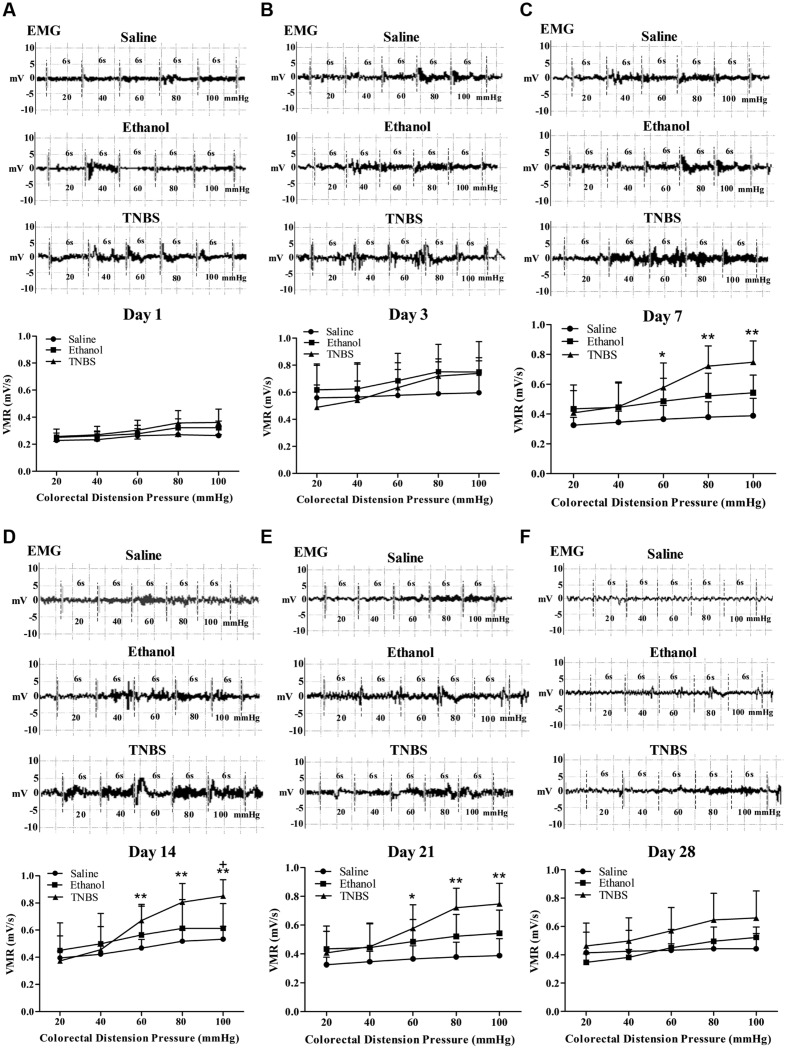
**Effects of TNBS administration on VH, measured by EMG of abdominal muscles’ VMR to CRD pressure.** Representative EMG traces and VMR to CRD at day 1 **(A)**, 3 **(B)**, 7 **(C)**, 14 **(D)**, 21 **(E)**, and 28 **(F)**. ^∗∗^*P* < 0.01, ^∗^*P* < 0.05; TNBS group/Ethanol group vs. Saline group. ^+^*P* < 0.05; TNBS group vs. Ethanol group. Kruskal–Wallis analysis followed by Mann–Whitney *U*-test.

The EMG of abdominal muscles’ was recorded for assessment of VH (**Figures 6A–F**). There was no change in the magnitude of the VMR between the saline- and ethanol-treated rats during the experiment. There was no difference (*P* > 0.05) in VMR with 20, 40, 60, 80, and 100 mmHg CRD pressures among the three groups at day 1 and 3. Compared with the saline rats, the TNBS-treated rats demonstrated higher (*P* < 0.05) VMR with 60, 80 and 100 mmHg CRD pressures at day 7 to day 21. The rats in the TNBS group demonstrated increased (*P* = 0.041) VMR with 100 mmHg of CRD at day 14 compared with the ethanol group. No difference (*P* > 0.05) was found in VMR with 20, 40, 60, 80, and 100 mmHg CRD pressures among the three groups at day 28.

### Correlation between Total VMR and CGRP Level

In TNBS-treated rats, the total VMR did not correlate (*P >* 0.05) with CGRP level at day 1 and 3. The correlations were observed between total VMR and CGRP level in TNBS-treated rats at day 7(*r* = 0.740, *P* = 0.036), 14 (*r* = 0.789, *P* = 0.020), and 21 (*r* = 0.736, *P* = 0.037). No correlation (*P >* 0.05) was found between total VMR and CGRP level in TNBS treatment at day 28 (**Table 2**).

**Table 2:** The correlation between CGRP level in DRG and total VMR in TNBS-treated rats.

Days	CGRP-IR	Total VMR (30 s)	Correlation coefficient	Significant level
1	16.54 ± 2.61	46.60 ± 10.80	*r* = 0.348	*P* = 0.398
3	19.87 ± 5.18	93.73 ± 18.13	*r* = 0.224	*P* = 0.593
7	28.22 ± 3.40	87.17 ± 21.33	*r* = 0.740^∗^	*P* = 0.036
14	62.27 ± 5.14	93.08 ± 22.20	*r* = 0.789^∗^	*P* = 0.020
21	20.85 ± 3.16	87.17 ± 21.33	*r* = 0.736^∗^	*P* = 0.037
28	11.28 ± 0.66	85.02 ± 25.77	*r* = 0.325	*P* = 0.431


*Data are expressed as Mean ± SD. ^∗^Pearson correlation between CGRP-IR and total VMR is significant at the 0.05 level (2-tailed).*

## Discussion

Crohn’s disease is a chronic IBD that can occur in any part of the gastrointestinal tract, but most frequently in the ileum. Currently the understanding of the mechanism underlying hypersensitivity of CD has been seriously hindered by a lack of animal models that faithfully resemble this disease of humans. A few researchers have used TNBS to induce ileal inflammation for physiological and pharmacological studies. O’Hara et al. (2007) injected TNBS (0.5 ml; 30 mg/ml in 30% ethanol) into the ileum of guinea pigs and observed a severe inflammation at 3 days. Moreels et al. (2001) injected 30 mg TNBS (dissolved in 0.25 ml of 40% ethyl alcohol) into rats’ ileum and found a marked inflammatory reaction occurred at 36 h and lasted up to 7 days. Nurgali et al. (2007, 2011) and Pontell et al. (2009) administered TNBS (30 mg/kg body weight in 1 ml of 30% ethanol) into the ileum of guinea pigs and observed the clinically as well as histologically inflammatory evidences up to 7 days. These studies showed a short duration of ileitis induced by TNBS. Shibata et al. (1993) injected 1 g TNBS (dissolved in 20 ml in 50% ethanol) into canine ileum, and induced severe mucosal lesions characterized by extensive ulcers and granulomas and lymphocyte infiltration for 2 months. It is seen that the severity and duration of TNBS-induced ileitis are dose- and concentration- dependent. In the present study, 80 mg/kg body weight TNBS in 30% ethanol caused weight loss, lethargies and bloody diarrhea, and histopathologically apparent changes characterized by hemorrhagic foci, mucosal necrosis, granulomas, distorted villi, diffuse infiltration of neutrophils and lymphocytes in lamina propia, and thickened ileal wall in rats. These symptoms and pathological changes in our study are similar to the macroscopic and microscopic findings observed in human CD. According to DAI scores, macroscopic, and microscopic changes, the TNBS-induced ileitis in our experiment occurred at 3 days, reached maximum at 7 days, persisted up to 3 weeks and healed spontaneously at 4 weeks. This dose and concentration of TNBS was found to be suitable for inducing subacute and chronic ileitis in rats. Besides, some other factors may also be responsible for discrepancies in pathological changes of TNBS-induced ileitis. Intestine emptying and peristalsis can reduce the contact time of TNBS with the ileal mucosa. Merritt et al. (2002) administered 0.5–1 g TNBS (dissolved in 33 to 75% ethanol) to porcine ileum with or without it confined to the lumen, and observed significant multifocal necrosis and ulceration in the pigs with the instillate was confined to the distal ileum, but did not in the pigs in which the instillate was not held within an ileal segment. The study of Merritt et al. (2002) did not show the resultant pathological changes because their experiment ended at day 7. We milked free of the gut content, occluded approximate ileal fragment with thumb and forefingers for 5 min to confine the injected TNBS solution. The TNBS injection induced apparent ileal lesions for 21 days. Granulomas are features of CD and have been described in approximately 50% of rats with TNBS colitis. Epidemiology suggests some relationship between the establishment of gut flora and the risk of developing inflammatory granulomas. In animal model, broad-spectrum antibiotics reduce the bacterial load and militate against gut inflammation. Brenna et al. (2013) found that TNBS solution (0.6 ml; 30 mg/ml in 50% ethanol) did not induce granuloma in colonic mucosa of rats at 12 days after TNBS administration, and attributed it to the intestinal bacterial reduction due to the sterile animal housing facilities. The granuloma development in the present study may be due to a certain number of microorganisms existing in the ileum (Duval-Araujo et al., 2007) and the possibility of their proliferation under the pathological condition.

Several studies have shown that patients with IBD demonstrate abdominal hypersensitivity to nociceptive stimuli applied to visceral tissues, and that there is discrepancy between the intensity of gastrointestinal symptoms such as pain or discomfort and the severity of mucosal lesions. Some animal models have developed inflammatory lesions in the ileum that are similar to human CD (Tanaka and Sugie, 2013). However, no any studies confirm whether or not VH exists. Adam et al. (2006) investigated various degrees (mild, moderate, and severe) of colitis induced by different concentrations (0.2, 0.4, and 0.8 ml; 5 mg/kg body weight in 50% ethanol) of TNBS, and observed the resultant visceral hyperalgesia through assessment of VMR to the mechanical CRD. They found that visceral hyperalgesia and its intensity are inflammation-severity dependent; moderate and severe inflammations (mild inflammation with repeated colorectal distention) evoked visceral hyperalgesia. Therefore, severity of mucosal inflammation could be used as a predictor for alterations of visceral sensory function. Several studies (Traub, 2000; Kyloh et al., 2011) indicated that the VH from the rectum is mediated via both thoracolumbar and lumbosacral spinal afferents pathways, whereas VH from upper colon or ileum is primarily mediated via thoracolumbar spinal afferents pathways. Because there is a convergence of nociceptive afferents from small and large intestines on the spinal neurons, CRDs can be used as stimuli to assess VH produced in TNBS-ileitis. In the present study, the rats with ileitis exhibited significant VMR to CRD at day 7 to day 21, demonstrating that VH did accompany TNBS-induced ileitis. The hypersensitivity reactions observed in this study might be evoked due to sensitization of both thoracolumbar and lumbosacral spinal afferents pathways, because the TNBS injection into the terminal ileum played role for sensitization of the thoracolumbar spinal afferents, while CRD resulted sensitization effects on the lumbosacral spinal afferents.

Mucosal mast cells are located in close proximity to the enteric sensory nerves throughout the gut and their activation or degranulation in response to stimuli release inflammatory mediators that can excite the sensory nerves (Stevens and Austen, 1989; Bueno et al., 1997). Ohashi et al. (2008) injected TNBS (50 mg/1.5 ml/kg body weight) into the colon of wild rats and mast cell knockout rats, and reported mucosal necrosis, extensive inflammatory cells infiltration in colonic mucosa of both wild rats and mast cell knockout rats. Interestingly, the TNBS-treated wild rats revealed VH but mast cells knockout rats failed to elicit VH in response to CRD. In the current experiment, the ileal mucosal mast cells increased in TNBS- or ethanol-treated rats at day 3 and in TNBS-treated rats at day 7, which resembles the markedly increased ileal mucosal mast cells in CD patients (Gelbmann et al., 1999). Studies showed that in biopsies obtained from patients with IBDs, the number of inflammatory and immune cells (e.g., mast cells, neutrophils, *T*-lymphocytes, and macrophages) were increased, and thereby activated inflammatory cells resulted in a release of certain mediators such as cytokines, proteases, etc., which affected the secreto-motor response of the gut and abnormal bowel functions (Weston et al., 1993; Chadwick et al., 2002; Barbara et al., 2004). MPO is mainly present in neutrophils and its oxidative products are involved in numerous processes of tissue damage in inflammatory pathology. In the TNBS-induced ileitis, MPO concentration was reported to increase during acute phase of the inflammation. In our study, it was increased at day 1 to day 14, which is similar to the previous report (Martinolle et al., 1997). It has been demonstrated that inflammatory cytokines have a critical role in the development of VH. In IBD and experimental colitis, monocytes in blood are recruited to the mucosa and differentiate into activated macrophages that produce proinflammatory cytokines, such as TNF-α, IL-1β, and IL-6 (Maloy and Powrie, 2011; Bain et al., 2013). It was reported that peripheral blood mononuclear cells from diarrhea-predominant IBS patients displayed elevated levels of TNF-α, IL-1β, and IL-6 (Liebregts et al., 2007). Adam et al. (2006) measured the serum IL-6 level in rats with TNBS-induced colitis, and found that the increased serum IL-6 was associated with increased VMR to CRD. However, the relationship between cytokine levels in inflamed ileal tissues and hypersensitivity has not been reported. In our study, TNF-α and IL-6 concentrations markedly increased at day 1 to 7 while IL-1β concentration was upregulated at 1 to 14 days. The three cytokines reached their peak levels at day 1 to day 3, which was earlier than the ileitis at day 7 and the VH at day 14. These findings ascertain the role of TNF-α, IL-6, and IL-1β in the initiation and maintenance of the ileitis and the VH. In present study, the increased VMR to graded CRD (20–100 mmHg) did not correlate with ileal IL-6 level (data not shown). Adam et al. (2006) reported that different concentrations of TNBS (0.4 and 0.8 ml; 5 mg/kg body weight) induced a moderate colitis followed by a biphasic change of visceral hyperalgesia in rats; the hyperalgesia was observed at day 3, but disappeared at day 14, and occurred again at day 28 to day 42 after TNBS administration. Feng et al. (2012) used TNBS (0.2 ml, 200 mg/kg body weight), intracolonally and observed severe colitis followed by significant VMR to CRD at day 7 to 14. The discrepancy in onset time and duration of VH between the reports by Adam et al. (2006) and Feng et al. (2012) may be due to different doses of TNBS. In our experiment, we found that TNBS (0.6 ml, 80 mg/kg body weight) induced a moderate ileitis and VH occurred at day 7 to 21, which is similar to the report by Feng et al. (2012).

Bernstein et al. (1996) presented the evidence of reduced sensitivity and autonomic reflex responses to rectal distension in ileal CD patients. Chang et al. (2000) reported that the colonic hypersensitivity is generally absent in patients either with UC in quiescent or mildly active phases of the IBDs. However, a study of Faure and Giguere (2008) in small population of pediatric CD patients coexisting functional GI disorders reported rectal hypersensitivity in 87.5% CD patients with low rectal sensory thresholds for pain compared with healthy controls. This discrepancy may be due to region-specific degree or duration of bowel inflammation. Annahazi et al. (2009) infused fecal supernatants from diarrhea-predominant IBS and UC patients into the colon of mice and demonstrated VH due to release of serine-protease (activator of protease activated receptor-2, PAR-2) and hyposensitivity because of released cathepsin-G (activator of PAR-4), respectively. Further explanation for the visceral sensitivity in CD could involve central sensitization (Bernstein et al., 2002; Van Oudenhove et al., 2004) or descending bulbospinal inhibition of sacral dorsal horn neurons in response to chronic intestinal tissue irritation (Bernstein et al., 1996). In our experiment, VH resolved in TNBS-treated rats at day 28, which may be caused by compensatory inhibitory mechanisms, counteracting VH.

Although the causes of VH are known to include some triggering events such as inflammation, trauma, or environmental stress, little is known about the mechanism underlying VH. TNBS-Ethanol combination works to cause tissue damage and induce intestinal inflammatory cell infiltration, release MPO, tryptase resulting activation of PAR-2, and cytokines (TNF-α, IL-6, IL-1β, etc.) in the compromised intestinal tissues. The interaction of these cytokines with inflammatory cells, mast cell, lymphocytes, and enteric plexuses sensitizes nociceptive afferent nerve terminals that are distributed to the ileum. PAR-2 activation leads to sensitization of transient receptor potential vanilloid subtype-1 receptors and triggers the release of SP and CGRP (Adam et al., 2006). Several studies highlighted the CGRP involvement in the visceral pain models (Friese et al., 1997; Plourde et al., 1997; Gschossmann et al., 2001). It has been reported that there is a remarkable change in expression of SP and CGRP in response to inflammation within DRG neurons as well as spinal neurons (Miampamba and Sharkey, 1998; Suckow and Caudle, 2009), which ultimately play an important role in neurogenic inflammation and hyperalgesia (Gschossmann et al., 2001; Sun et al., 2004). In this study, TNBS treatment remarkably increased the CGRP expressions in the DRG of rats at day 7 to 21, which correlated with the VH. Sustained depolarization of the nerve terminals would prolong firing, resulting in increased afferent traffic to the specific neurons in the spinal cord where excitatory signals are transmitted by the relay neurons to the higher central nerve system (Moore et al., 2002). Therefore, our model is useful to study the specific central regulating mechanism underlying VH caused by the ileitis. The VH maintained at a stable and continuous level in this study, which will be beneficial for studying the specific central regulating mechanism underlying VH caused by the ileitis.

## Conclusion

2,4,6-trinitrobenzene sulfonic acid injection into the terminal ileum caused weight loss, lethargies and bloody diarrhea and, successfully induced transmural ileitis supported by pathological changes and the releases of cytokines in rats, which is similar to human CD. The significantly increased CGRP expressions level in DRG correlated with increased VMR to CRD in TNBS-induced ileitis at day 7 to 21, which unravels the existence of VH. These data suggest its potentiality to probe into the pathogenesis of inflammatory ileal diseases and VH as well as for evaluating the effectiveness of new therapeutic agents.

## Author Contributions

MS, WJ, and M-XD contributed to conception and design of the study. MS, WJ, HJ, and AT performed animal experiments, collected samples and accomplished the laboratory investigations. LC, WJ, HJ, and AT performed acquisition of data. MS, LC, and WJ conducted data analysis and interpretation of data. MS, LC, AT, and HJ drafted the manuscript. M-XD, MS, LC, AT, HJ, and WJ revised the manuscript. All authors read and approved the final manuscript.

## Conflict of Interest Statement

The authors declare that the research was conducted in the absence of any commercial or financial relationships that could be construed as a potential conflict of interest.

## Abbreviations

CD: Crohn’s disease
CGRP: calcitonin gene-related peptide
CGRP-IR: calcitonin gene-related peptide-immunoreactive
CRD: colorectal distension
DAI: disease activity index
DRG: dorsal root ganglia
ELISA: enzyme-linked immunosorbent assay
EMG: electromyography
IBD: inflammatory bowel disease
IBS: irritable bowel syndrome
IL-1β: interleukin-1 beta
IL-6: interleukin-6
MPO: myeloperoxidase
TNBS: 2,4,6-trinitrobenzene sulfonic acid
TNF-α: tumor necrosis factor-alpha
UC: ulcerative colitis
VH: visceral hypersensitivity
VMR: visceromotor responses
